# A loss-based patch label denoising method for improving whole-slide image analysis using a convolutional neural network

**DOI:** 10.1038/s41598-022-05001-8

**Published:** 2022-01-26

**Authors:** Murtaza Ashraf, Willmer Rafell Quiñones Robles, Mujin Kim, Young Sin Ko, Mun Yong Yi

**Affiliations:** 1 Department of Industrial and Systems Engineering, Graduate School of Knowledge Service Engineering, Korea Advanced Institute of Science and Technology, Daejeon, South Korea; 2 Pathology Center, Seegene Medical Foundation, Seoul, South Korea

**Keywords:** Cancer imaging, Gastric cancer, Data processing, Machine learning, Computer science, Software

## Abstract

This paper proposes a deep learning-based patch label denoising method (*LossDiff*) for improving the classification of whole-slide images of cancer using a convolutional neural network (CNN). Automated whole-slide image classification is often challenging, requiring a large amount of labeled data. Pathologists annotate the region of interest by marking malignant areas, which pose a high risk of introducing patch-based label noise by involving benign regions that are typically small in size within the malignant annotations, resulting in low classification accuracy with many Type-II errors. To overcome this critical problem, this paper presents a simple yet effective method for noisy patch classification. The proposed method, validated using stomach cancer images, provides a significant improvement compared to other existing methods in patch-based cancer classification, with accuracies of 98.81%, 97.30% and 89.47% for binary, ternary, and quaternary classes, respectively. Moreover, we conduct several experiments at different noise levels using a publicly available dataset to further demonstrate the robustness of the proposed method. Given the high cost of producing explicit annotations for whole-slide images and the unavoidable error-prone nature of the human annotation of medical images, the proposed method has practical implications for whole-slide image annotation and automated cancer diagnosis.

## Introduction

One challenging application of artificial intelligence (AI) is diagnosing heterogeneous diseases that can lead to death in humans. Cancer, for example, is such a disease and one of the leading causes of death worldwide, ranking 2nd in deaths per year in the United States^1^. The World Health Organization reported that the global burden of cancer is expected to grow by 29.4 million new cases by 2040^2^. To diagnose the existence of cancer, whole-slide images are commonly processed by a pathologist. It has been reported that pathologists are often susceptible to errors based on different pathologists, specimen types, and diagnoses, and Type-I and Type-II errors occur in 6% and 33% of cases, respectively^3^.

The computer-aided analysis of whole-slide images is a complicated process due to the nature of a cell’s biological morphology, which conventional machine learning methods may fail to generalize, even when coupled with handcrafted feature extraction^4^. With recent advancements in convolutional neural network (CNN)-based computer vision applications, it is believed that AI can enable automated diagnoses of whole-slide images^5^. CNNs can extract features automatically, but their data-hungry nature requires the labeling of a large number of whole-slide images. Additionally, obtaining comprehensive annotations for whole-slide images can be difficult for various reasons, such as lack of prior experience, human bias, and technical issues, and the time and availability of professional pathologists are often limited. To produce training data for automated systems, pathologists annotate abnormal regions in whole-slide images, and other regions are automatically considered benign (negative).

**Figure 1:**
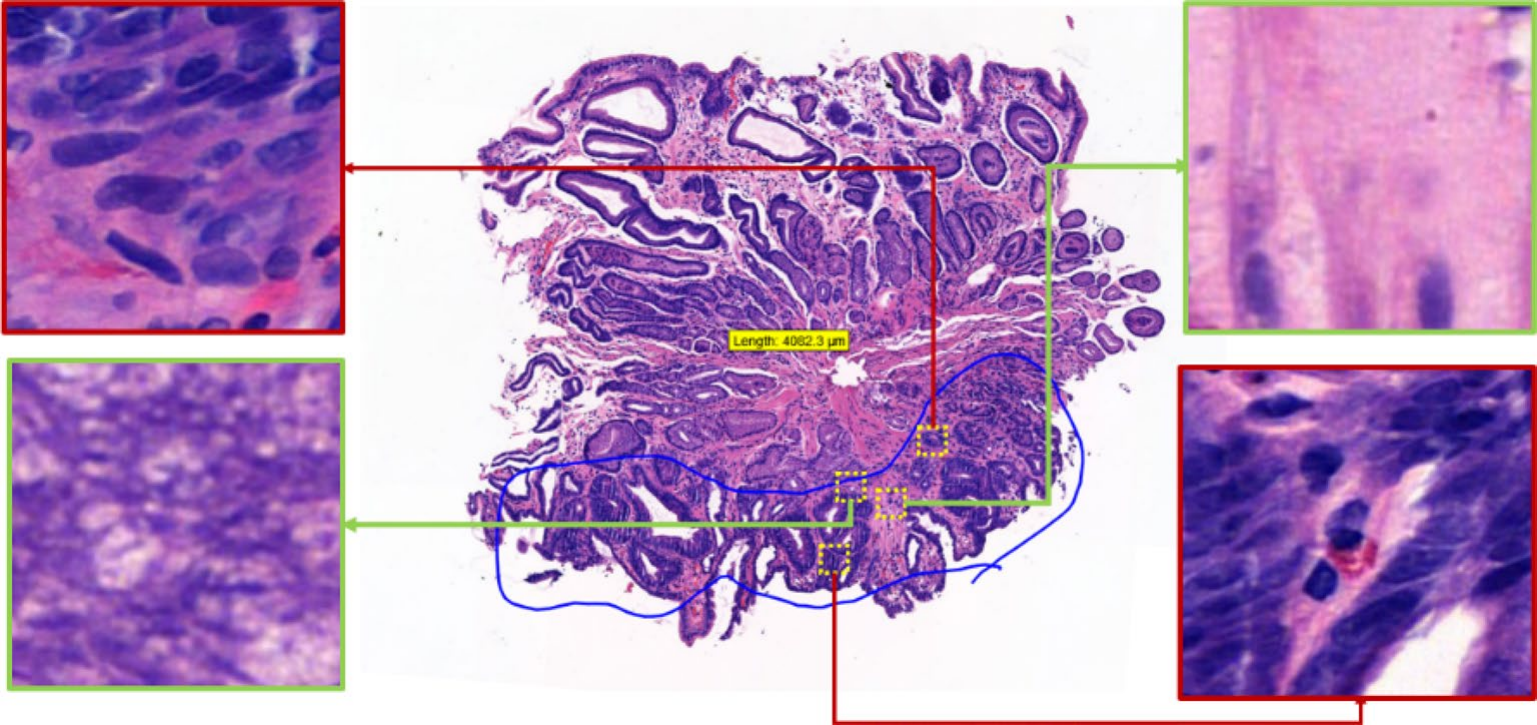
The portion of the tissue circled in blue is a dysplasia annotation by a professional pathologist. The red zoomed-in regions are abnormal (true positive) regions within the annotation and the green zoomed-in regions are normal, benign (false positive) regions within the annotation.

Malignant annotations can incorporate some of the small areas of benign cells or different kinds of pathological findings, such as atypical cells and inflammation, as illustrated in Fig. 1. Hence, these annotations can introduce patch-based label noises (e.g., false positives); it is very difficult, if not impossible, for pathologists to precisely mark each abnormal region with a pixel-by-pixel approach. A frequently adopted practice is to collaborate with multiple medical experts and seek their inputs on unreliable annotations for improved accuracy and consistency. Nevertheless, this additional measure does not guarantee 100% accuracy and can still lead to bias and time constraint issues.

A review of the literature has revealed that label noise modeling is generally based on distinguishable object datasets such as MNIST^6^, CIFAR^7^, and ImageNet^8^. Medical data, such as digital pathology data, have rarely been used in this context^9^. Pathologists mainly analyze whole-slide images to identify abnormal cells. A whole-slide image is a gigapixel image, and such images often cannot be processed with a CNN. Thus, researchers divide whole-slide images into small patches. Those small patches can easily incorporate some of the normal regions (false positives), adding label noise to the input data. Most of the time, digital pathology classification tasks have ignored patch-based label noise, resulting in low accuracy with many Type-II errors (additional details in the next section). To overcome this critical problem, this study presents a simple yet effective method for noisy patch classification to enhance the automated analysis of whole-slide images.

Motivated by the aforementiond research need, the objective of this study is to design and evaluate a patch-based label noise abstaining method that allows CNNs to produce better classification results. The proposed method avoids the need for extra layers in the neural network and does not require a set of verified annotations, as is required in other approaches^9,10^. The findings from the present study can serve as a basis for refining digital pathology training data. Specifically, the contributions of this study are threefold. First, this is one of the first studies to propose a CNN-based label denoising method for whole-slide images that requires neither additional learnable parameters nor a set of precise annotations for the training process. Second, we established a new multiclass dataset for stomach whole-slide images and rigorously evaluated a CNN for classification; the robustness of the proposed approach was also confirmed at different noise levels using a publicly available dataset. Third, to the best of our knowledge, our study is one of the first endeavors to evaluate and compare state-of-the-art label denoising methods based on pathological images.

## Background

Computer vision has benefited from CNNs, which provide effective architectures for object detection^11^, face recognition^12^, autonomous vehicles^13^, and medical applications^14^. CNNs became popular after achieving state-of-the-art accuracy in 2012^15^ and winning the ImageNet challenge^8^. Later, several popular CNN schemes, such as the Visual Geometry Group (VGG) network^16^, Inception (GoogleNet)^17^, ResNet^16^, and DenseNet^18^, were introduced, and they have continuously outperformed existing methods in the ImageNet challenge. Recently, these schemes have been further enhanced and extended to address various practical problems^19–21^.

CNNs have been applied in medical imaging diagnostic systems^22^. In medical image analysis, CNNs have improved the detection, classification, and segmentation of manifold abnormities^14^. In particular, CNNs play an important role in cancer analysis, including in skin^23^, breast^24^, lung^25^, and endoscopy classification^26–30^. The availability of big data in the medical domain has enabled researchers to apply deep learning methods, which often require huge amounts of data to properly learn the underlying mechanisms and provide promising results. Moreover, compared to other data types, clinical data require more labeling effort from medical practitioners, who are typically highly trained, expensive, and overworked. One potential solution to this problem is to employ a nonexpert labeling approach based on image data^31^. However, this approach may exacerbate the label noise problem, thus limiting the practicality of deep learning-based diagnostic systems. Noisy data (or label noise) not only affect the performance of a machine learning model but also produce biased results^32–35^. To mitigate such label noise, deep learning models need to be trained with large amounts of correctly labeled data^36^; however, acquiring large amounts of precisely labeled data is challenging^37^.

A review of the existing literature was performed to identify the different methods used to mitigate label noise in different domains using CNNs. Some studies, for example, introduced an extra layer before or after a softmax layer during modeling for the processing of noisy labels^38,39^. These studies evaluated noise recognition layers based on the Google Street View house number dataset^40^, the Tiny Image dataset^41^, and MNIST^4^. This method can learn the distribution of noisy labels, but computational efficiency is low because the model needs to learn several extra parameters. Goldberger and Ben-Reuven proposed a training method by adding a softmax layer with expectation maximization^42^ to a CNN architecture; notably, the result of the final layer of the network is used to predict the probability that a label is incorrect or correct^43^. However, expectation maximization has convergence issues, and adding an extra layer along with expectation maximization would further aggravate the convergence problem. Another method involves semi supervised learning with a small set of verified labels; these verified labels can be used to transfer knowledge to incorrect labels^44^. The use of a small set of verified labels can enable a CNN to learn the relevant distribution from confirmed labels. However, verified labels, even small sets of them, can be difficult to arrange when the data are obtained from a public repository or released by an organization.

Deep learning models that can limit label noise in the medical domain are still in the early stages of development, and only a few studies have focused on label noise in the medical field. For instance, Dgani et al.^45^ proposed an incorrect label correction method using deep learning for breast microcalcifications; they used a noisy channel as part of a deep learning model to learn the noisy label distribution and added an extra layer in addition to the softmax layer^39^, which enabled their model to learn noise representations as a part of the CNN training process. Recently, using a small clean dataset of whole-slide images of pancreatic cancer, Le et al.^46^ predicted the distribution of noisy labels from imbalanced data; notably, few cleaned samples were available, and noisy data were abundant. Karimi et al.^9^ surveyed several methods for diagnosing diseases based on the detection and classification of abnormalities; they also evaluated interobserver label noise removal methods based on prostate cancer images. In their study, they focused on annotations from six different pathologists and aggregated their annotations. However, it is difficult to coordinate and afford large numbers of expert pathologists. Gehlot et al.^47^ proposed an unsupervised approach for avoiding label noise and obtained encouraging results based on different datasets. Their method leverages a dual-branch architecture with a given threshold to predict label noise when the results of both branches differ. In this architecture, one branch uses project loss, as proposed by Gehlot et al., and the other uses cross-entropy. The benefit of such an approach is that it provides diverse predictions similar to those produced with ensemble modeling. Nevertheless, this method requires multiple loss functions, which reduces interpretability. Moreover, the final decision, which is based on a coupled classifier or an ensemble decision, is often complex.

In summary, there is a need to develop a method that can automatically detect and eliminate noisy patches from whole-slide image annotations to ultimately produce accurate classifications of cancer. Most previous research was based on benchmark datasets involving digits, objects, and places; however, methods for noisy medical image data are still in the initial development phase. Several researchers have proposed modeling techniques by adding extra layers to CNNs, and the use of small sets of precise annotations has also been considered. Nevertheless, all these techniques are limited by time and computational constraints. To overcome these limitations, our study proposes and evaluates a novel method for denoising the patches extracted from whole-slide images and produces improved classifications of cancer.

## Methods

### Stomach pathology patch dataset

Stomach cancer is one of the leading causes of death among many other types of cancers and ranks 5th in new cases globally each year^48^. In 2021, the American Cancer Society estimated that 26,560 new cases of stomach cancer occurred in the United States^49^. The World Cancer Research Fund reported that South Korea had the highest rate of stomach cancer worldwide in 2018^50^. Given this prevalence, whole-slide images of stomach cancer were collected from one of the largest medical foundations in South Korea. The whole-slide images contain information about suspected regions obtained based on the extraction of gastric endoscopic biopsy specimens. The slides were stained with a hematoxylin and eosin staining process. All of the slides were reviewed and annotated by two pathologists who worked on separate sets of slides initially but examined each other’s work for verification.

**Figure 2:**
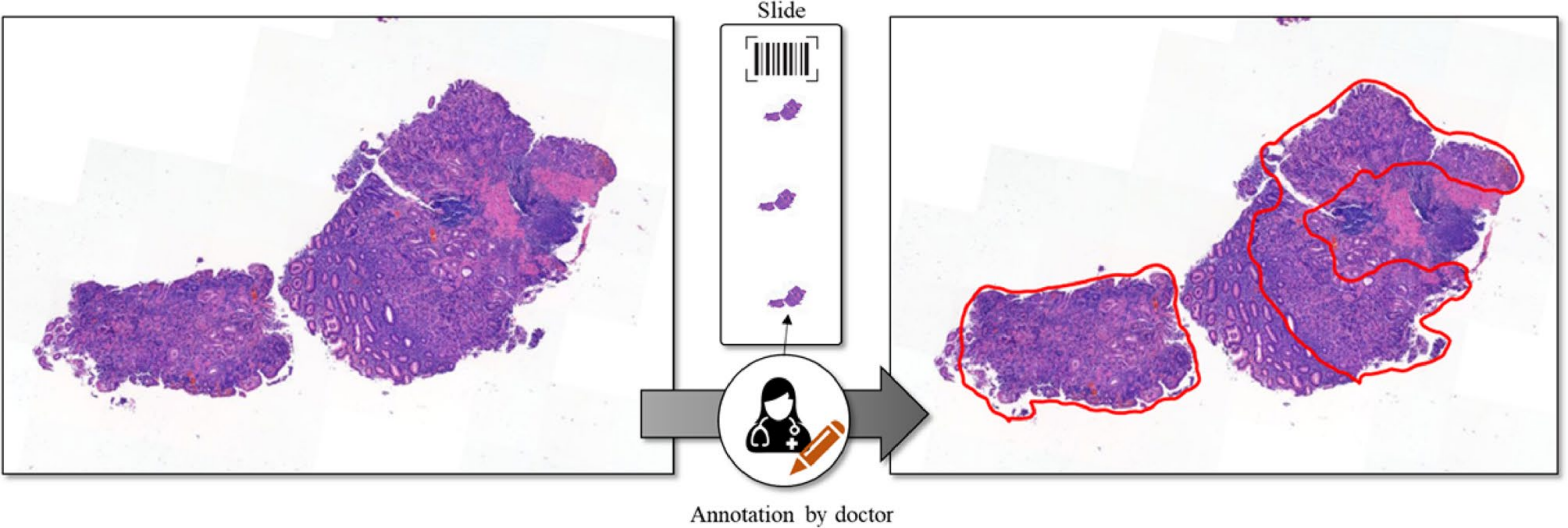
Example of hematoxylin and eosin-stained raw (left) and annotated (right) whole-slide images.

The data were collected by the Seegene Medical Foundation in South Korea, and their use for research was approved by the Institutional Review Board (Approval # SMF-IRB-2020–007) of the organization as well as by the Institutional Review Board (Approval # KAIST-IRB-20–379) of Korea Advanced Institute of Science and Technology (KAIST). Informed consent to use the tissue samples for clinical purposes was obtained from the medical foundation’s designated collection centers. All experiments were performed in accordance with the relevant guidelines and regulations provided by the two review boards. All patient records were completely anonymized, and all the images were kept and analyzed only on the company server. A sample set of an original slide and the corresponding annotated slide is presented in Fig. 2, and the details of data acquisition are presented in Table 1.

**Table 1:** Data acquisition details.

Parameter	Details
Thickness of section	3–4 µm
Staining method	Hematoxylin and eosin
WSI scanner model	Panoramic Flash 250 III
Sensor resolution	200×
Number of pathologists for annotation	2

## Details of the classes of stomach pathology patches

**Figure 3:**
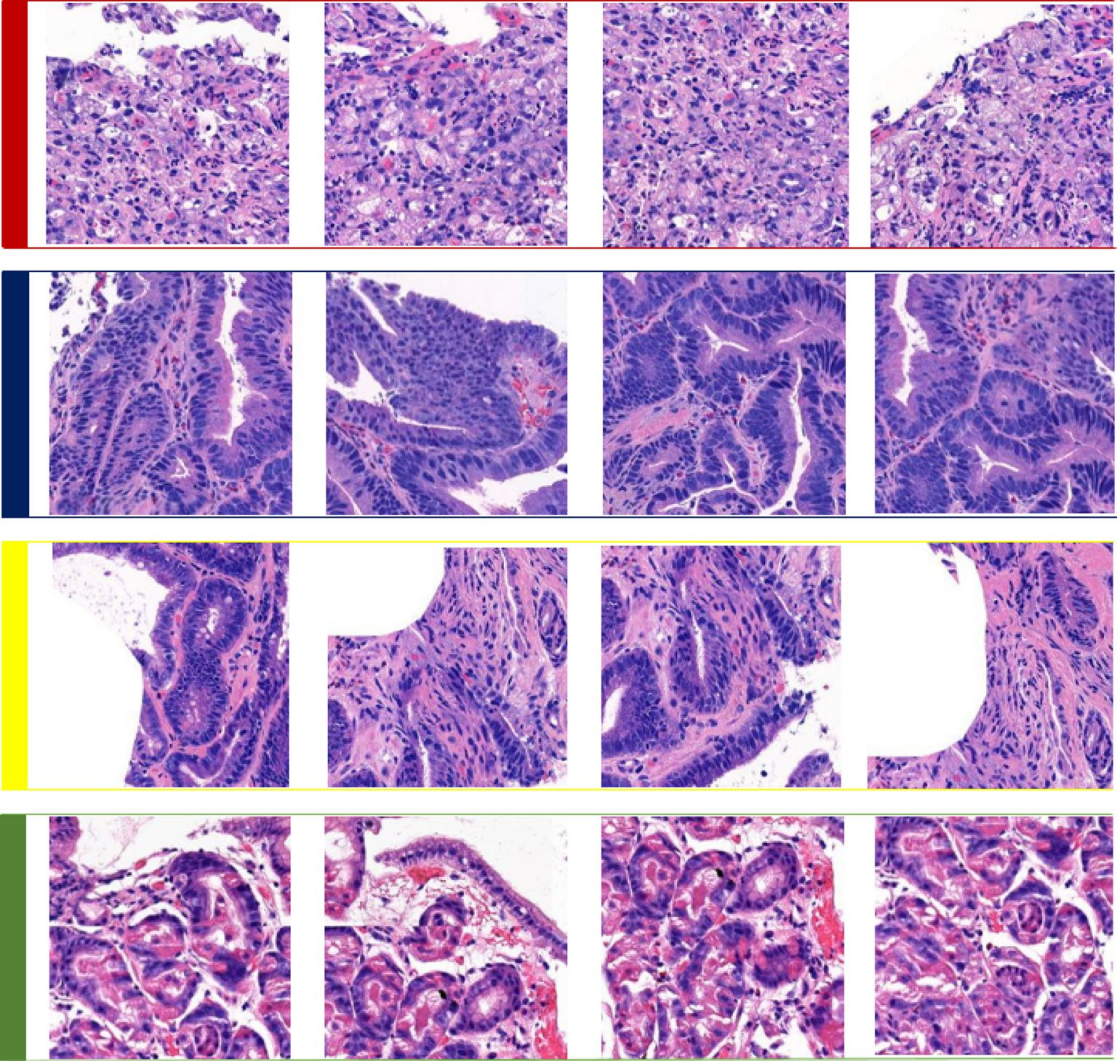
Four types of pathologic classes in whole-slide images of the stomach: red (1st row), navy blue (2nd row), yellow (3rd row) and green (4th row) annotated patches represent malignant, dysplasia, uncategorized, and benign classes, respectively.

Four classes of pathologic findings, namely, malignant, dysplasia, uncategorized, and benign classes, were analyzed in this study, and corresponding samples are shown in Fig. 3.

### Malignant

Diagnosed as malignant neoplasm, including adenocarcinoma, suspicious for (s/f) adenocarcinoma, suggestive of (s/o) adenocarcinoma, (s/f, s/o) high-grade lymphoma, and any other (s/f, s/o) carcinoma or malignant neoplasm.

### Dysplasia

Diagnosed as dysplasia, including (s/f, s/o) tubular adenoma with dysplasia of any grade.

### Uncategorized

The remaining lesions that do not fall under the aforementioned three classifications; for example, atypical glandular proliferation of undetermined significance, (s/f, s/o) neuroendocrine tumors, submucosal tumors, (s/f, s/o) low-grade lymphoma, and (s/f, s/o) stromal tumors, among others.

### Benign

Diagnosis of a nonneoplastic benign gastric mucosal lesion, including gastritis and polyps.

## Data preparation for stomach pathology patches

A whole-slide image can have a scale larger than 1 gigapixel. Due to computational resource limitations, CNNs cannot process such large inputs. Therefore, an open-source Python library '*OpenSlide*' was used to divide each whole-slide image into smaller patches. The patches were then processed to exclude the white areas of slides (i.e., parts without tissue). Each patch was then labeled with a slide number, patch position, and particular class. Considering the current direction of research regarding noisy label elimination, we divided the dataset into two parts: pilot data and baseline data. A small subset from the whole dataset was selected as the pilot dataset to determine the noisy patch data distribution. The baseline dataset was used for classification. The details of each dataset by class are shown in Table 2. Out of the total number of 905 baseline WSIs, we used 80% for training, 10% for validation, and 10% for testing.

**Table 2:** Information about the number of stomach whole-slide images (WSIs) for each data split.

		Classes			
		Malignant	Dysplasia	Uncategorized	Benign
Pilot WSIs		24	30	10	35
Baseline WSIs	Training	174	220	75	254
	Validation	22	27	10	32
	Testing	22	27	10	32

To ensure their independence, training, validation, and test data were separated at the patient level (i.e., whole slide). The number of patches, as shown in Table 3, varied based on different annotation sizes. There were more patches in the benign class than in the other classes because no annotation was required for benign tissue and we extracted patches from complete slides. In contrast, malignant, dysplastic, and uncategorized patches were smaller in number because they were extracted from annotated regions only.

**Table 3:** Information about the number of patches for each data split based on stomach whole-slide images.

		Classes			
		Malignant	Dysplasia	Uncategorized	Benign
Pilot patches		2172	2435	423	4890
Baseline patches	Training	26,855	21,881	8376	49,564
	Validation	2563	2324	1006	6476
	Testing	3078	2772	247	4588

### PatchCamelyon

**Figure 4:**
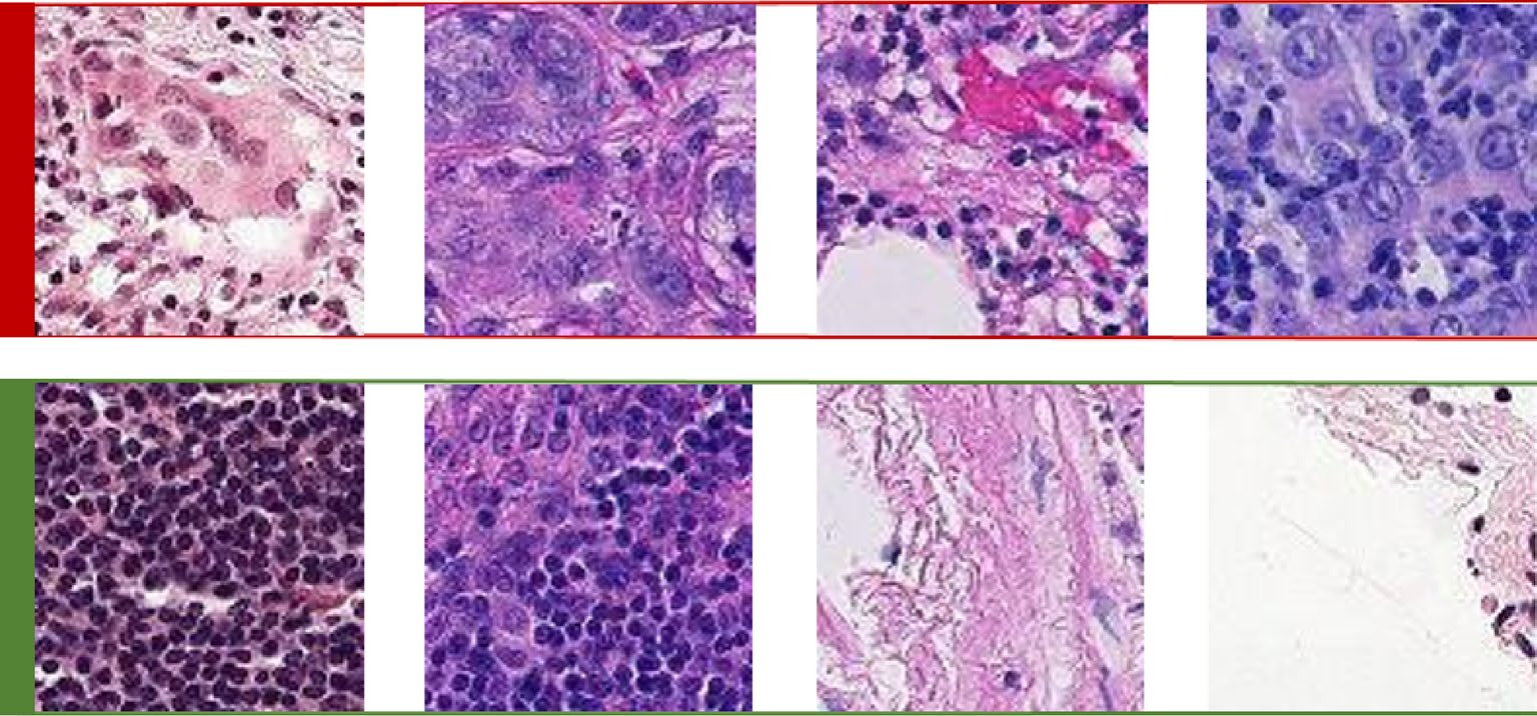
Two types of pathological findings for lymph node sections: red (1st row) and green (2nd row) annotated patches denote malignant and benign classes, respectively.

Given that the dataset described in the previous section cannot be shared for public use and to ensure the reproducibility of the results, we additionally use a publicly available dataset called PatchCamelyon^51^, which contains 327,680 pathological patches, in this study. Patches of size 96 × 96 were extracted from the histopathological scans of lymph node sections^52^. As shown in Fig. 4, each patch was annotated with a positive label (malignant) or negative label (benign), indicating the presence of metastatic tissue. Note that we ensured that there was no overlap in WSIs across the training, validation, and test splits to avoid any bias in model predictions. We also ensured that each split was equally balanced between positive and negative samples. Details on the number of patches by class are given in Table 4.

**Table 4:** Information about the number of patches in each data split for PatchCamelyon.

		Classes	
		Malignant	Benign
Patches	Training	131,072	131,072
	Validation	16,369	16,399
	Testing	16,377	16,391

## Model formulation

**Figure 5:**
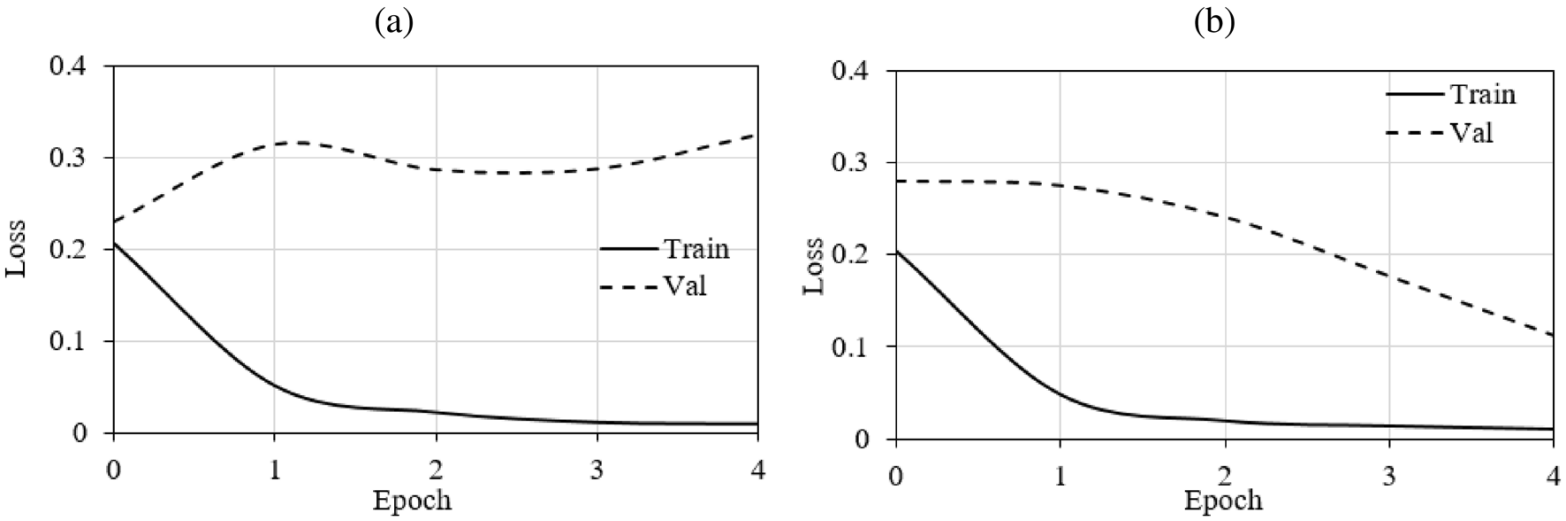
Training and validation loss of models with (**a**) noisy data and (**b**) cleaned data; *Train* training; *Val* validation.

Deep learning models tend to overfit when trained for a long time because of their tendency to memorize the data distribution. Although most of the features of a class exhibit the same data distribution, if there are some noisy labels, then the model may learn the characteristics of the corresponding features. Forced learning without noise can lead to overfitting. Images with label noise are associated with higher loss than are images with true labels, and based on this relation, our proposed method eliminates the patches with batch loss levels higher than the average loss. To compare the performance of the proposed method and the baseline method, Fig. 5 presents the training loss and validation loss of the models over the five initial epochs using both cleaned and noisy data. Notably, the model with noisy data (see Fig. 5a) experiences overfitting within the initial five epochs, and the proposed method (see Fig. 5b) avoids overfitting.

**Figure 6:**
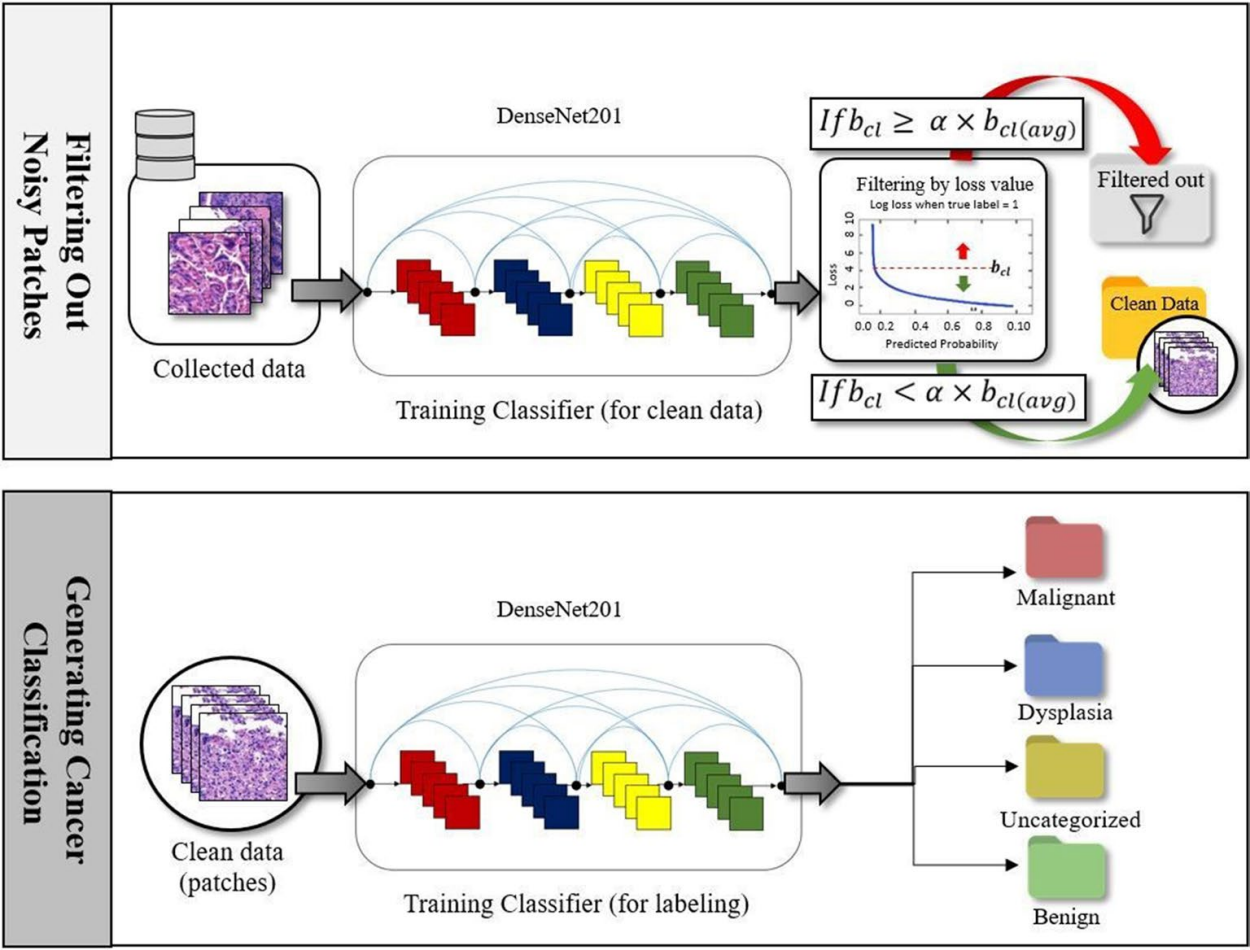
The second and third phases of the proposed *LossDiff* method.

Given a whole-slide image *X* marked with unavoidable noise introduced by human annotators, our goal is to accurately predict the type of disease *Y* by extracting useful features from a set of patches $P=\{{p}_{1},{p}_{2},{p}_{3},\dots {p}_{m}\}$ using a CNN. To achieve this goal, we propose a new whole-slide image classification method called *LossDiff*, which consists of three phases: (1) selecting an optimal CNN architecture, (2) filtering labeled noisy patches, and (3) performing cancer classification*.* The first phase involves identifying the most suitable underlying architecture of a CNN. As shown in Fig. 6, we filter and remove the patches with label noise by considering the average batch loss for correctly classified instances in the second phase and perform the classification of diseases based on the cleaned data using the CNN architecture in the third phase. The baseline modeling approach, which was used for performance comparison, does not include the second phase and uses the baseline dataset (i.e., no noisy patches removed) for the third phase.

### Selecting an optimal CNN architecture

We analyzed popular CNN models based on the baseline data to assess the performance of different types of architectures for whole-slide images. CNN architecture selection enables us to choose the best-suited CNN for pathological data. Various CNNs, namely, AlexNet, Inception, VGG, ResNet and DenseNet, were assessed in this study. These architectures have been trained on large sets of images from ImageNet (Deng et al., 2009) and training parameters are provided to help fine-tune the CNN models for other classification problems. We considered two approaches: fine-tuning the pretrained models and training the models from scratch on pilot data. The purpose of the performance comparison was to validate the use of a fine-tuning approach rather than training from scratch and selecting a baseline architecture. The benefits of fine-tuning based on limited data are generally acknowledged. However, some researchers, such as Raghu et al.^53^, have reported that there is little difference in fine-tuning and training from scratch. In our experiments based on stomach whole-slide images, there is a difference of approximately 3% between the results of these two approaches, as shown in Table 5. Due to time constraints, the stopping criterion of 30 epochs was the same for the two approaches.

**Table 5:** Preliminary study for selecting the final architecture (the accuracy of each architecture is reported as a percentage*).*

Architecture	Method	
	Fine-tuning pretrained model	Training from scratch
AlexNet	69.21	66.07
Inception	72.45	71.86
VGG	72.13	71.32
ResNet	73.09	70.09
DenseNet	73.38	70.78

Our preliminary results revealed that pretrained models perform better than models trained from scratch when whole-slide images are used. A brief summary of the comparison is presented in Table 5. We also found that the models that incorporate large numbers of layers with residual blocks perform better than other models. Table 5 shows that ResNet and DenseNet, which consist of residual blocks, outperform all the other architectures, and DenseNet is the best-performing architecture. Based on the preliminary results using stomach whole-slide images pilot data, we selected pretrained DenseNet (DenseNet-201) as the final architecture. The architecture selection was done on the stomach dataset only, and the same network type was then trained on the PatchCamelyon set.

### Filtering noisy patches

We propose a fast and efficient patch label denoising method for handling label noise. In this approach, we distinguish between correctly labeled patches and noisy patches. We first extract the patches $P$ from a whole-slide image using the *OpenSlide* library. These patches are then transformed into the input tensor of the model, and we optimize cross-entropy loss by training DenseNet for a specific number of epochs. After training the model for a specific number of epochs, we observe the loss (${b}_{l}$) based on the baseline dataset with label noise (${D}_{b}$). At this point, we keep a record of the loss results for correctly classified instances $y=\widehat{y}$ for each patch type $t$, where $y$ is the ground truth, $\widehat{y}$ is the model prediction, and $t \in \{D, M, N, U\}$. Given a batch $b$ of $m$ instances, the loss for a number of correctly classified instances can be defined as ${b}_{cl}=\left\{{l}_{c1},{l}_{c2}, {l}_{c3},\dots {l}_{cm}\right\}$, where ${l}_{cm}$ denotes the loss $l$ of $m$ correctly classified instances $c$. In addition, the loss for correctly classified instances and each patch type $t$ is tracked within a batch, and we monitor the average loss in the same way with the following equation: ${b}_{cl(avg)}=\left\{\left(\frac{{\sum }_{i=1}^{k}{l}_{c1}}{k}\right),\left(\frac{{\sum }_{i=1}^{k}{l}_{c2}}{k}\right),\left(\frac{{\sum }_{i=1}^{k}{l}_{c3}}{k}\right),\dots \left(\frac{{\sum }_{i=1}^{k}{l}_{cm}}{k}\right)\right\}$, where $k$ is the total number of training iterations for the model. To avoid filtering difficult cases, we introduce a threshold $\alpha$ that can be adjusted with respect to the data distribution. Mathematically, the abstaining condition can be formulated as

1 $${b}_{cl}\ge \alpha *\left(\frac{{\sum }_{i=1}^{k}{l}_{cm}}{k}\right).$$

Finally, we can formulate a function to produce the cleaned data ${D}_{c}$ and eliminate label noise as

2 $$f\left({D}_{b}\right)=\left\{\begin{array}{l}Remove p, {b}_{cl}\ge \alpha \times {b}_{cl\left(avg\right)} and y=\widehat{y}\\ Keep p, {b}_{cl}<\alpha \times {b}_{cl(avg)}\end{array}\right.$$

If the batch loss ${b}_{cl}$ is greater than the average batch loss ${b}_{cl(avg)}$ and the ground truth labels $y$ match the predicted labels $\widehat{y}$, then the model filters out the patches $p$. This process enables the model to generate cleaned data ${D}_{c}$ by reducing the effect of overfitting.
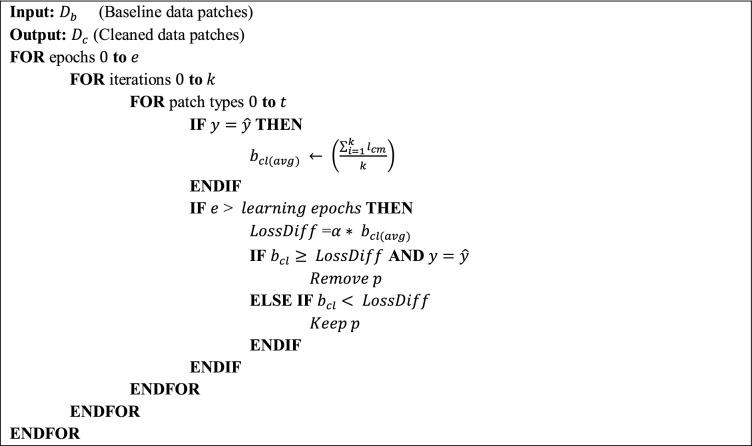


### Cancer classification

We selected the pretrained DenseNet for the classification of whole-slide images based on the preliminary results presented in Table 5. DenseNet uses residual connections so that each layer can receive additional inputs from all of the preceding layers in addition to the output of the previous layer. With this property, there are two main advantages of DenseNet: gradient flows are simple, and features are diverse. Multiple connections to the preceding layers enable the model to indirectly perform deep supervision and provide diverse features as inputs to each layer (see the original source^18^ for detailed information). The specific details of the DenseNet-201 model used in the experiments in this study are provided in Table 6.

**Table 6:** DenseNet-201 architecture details for the experiments.

Layers	Output Size	DenseNet-201
Convolution	112 $\times$ 112	7 $\times$ 7 convolution, stride 2
Pooling	56 $\times$ 56	3 $\times$ 3 max pooling, stride 2
Dense block (1)	56 $\times$ 56	$$\left[\begin{array}{ccc}1& \times & 1 conv\\ & & \\ 3& \times & 3 conv\end{array}\right]$$ $\times$ 6
Transition layer (1)	56 $\times$ 56	1 $\times$ 1 convolution
	28 $\times$ 28	2 $\times$ 2 average pooling, stride 2
Dense block (2)	28 $\times$ 28	$$\left[\begin{array}{ccc}1& \times & 1 conv\\ & & \\ 3& \times & 3 conv\end{array}\right]$$ $\times$ 12
Transition layer (2)	28 $\times$ 28	1 $\times$ 1 convolution
	14 $\times$ 14	2 $\times$ 2 average pooling, stride 2
Dense block (3)	14 $\times$ 14	$$\left[\begin{array}{ccc}1& \times & 1 conv\\ & & \\ 3& \times & 3 conv\end{array}\right]$$ $\times$ 48
Transition layer (3)	14 $\times$ 14	1 $\times$ 1 convolution
	7 $\times$ 7	2 $\times$ 2 average pooling, stride 2
Dense block (4)	7 $\times$ 7	$$\left[\begin{array}{ccc}1& \times & 1 conv\\ & & \\ 3& \times & 3 conv\end{array}\right]$$ $\times$ 32
Classification layer Final layer	1 $\times$ 1	7 $\times$ 7 global average pooling
		Softmax (2 | 3 | 4)

In the final layer, 2 refers to the malignant and benign classes; 3 refers to the malignant, dysplasia, and benign classes; and 4 refers to the malignant, dysplasia, uncategorized, and benign classes.

## Results

The proposed method was implemented in Python using ‘*PyTorch*’^54^, an open-source deep-learning library. The model was trained on a high-performance server equipped with an NVIDIA Titan XP GPU. The pretrained DenseNet-201 was used as the CNN architecture. Cross-entropy loss was optimized using the Adam optimizer^55^ with a learning rate of 0.001. The model was trained for 30 epochs with a batch size of 32. A data preprocessing pipeline was designed to enable the loading of whole-slide images and to filter and remove the patches without tissue regions. The data preprocessing pipeline uses the *OpenSlide* library^56^ and generates patches of the required size, which is 256 × 256 in this study. The proposed method, *LossDiff*, filters and removes suspicious patches, leaving fewer patches than in the baseline data. Therefore, to evenly compare the performance of different methods, we made the number of baseline and *LossDiff* test distribution patches equal using random sampling. Performances of the proposed model were assessed using: (a) accuracy, (b) a confusion matrix, (c) the area under the ROC curve, (d) the feature space visualization result using *t*-SNE, and (e) the results of a noise handling analysis based on a publicly available dataset. We also conducted the McNemar^57^ test to establish that the models trained on the cleaned data and on the baseline data are significantly different. All these analyses were performed in the ‘*scikit-learn*’^58^ Python library.

Note that the uncategorized class contained fewer whole-slide images than other classes due to the nature of the diseases considered. Thus, the performance of the model was assessed separately for ternary (malignant, dysplasia, and benign) and quaternary (malignant, dysplasia, uncategorized, and benign) classes. Binary class experiments were carried out on malignant and benign class data only. In a similar fashion for ternary class experiments, we have excluded uncategorized class data.

Furthermore, in the noise handling ability analysis, we selected the PatchCamelyon dataset because it uses magnification downsampling to 10 × from whole-slide images of 40 × magnification to increase the field of view. Expanding the field of view (i.e., by zooming out) eliminates the noise in baseline data and enables us to add a specific ratio of synthetic noise.

### Accuracy analysis

The accuracy of the proposed method, as reported in Table 7, can be obtained as follows:

**Table 7:** Accuracy comparison between the baseline and *LossDiff* results for ternary and quaternary classes.

Classes	Method			Malignant and benign (binary)	Malignant, dysplasia, and benign (ternary)		Malignant, dysplasia, uncategorized, and benign (quaternary)
Accuracy	Baseline (${D}_{b}$)			94.73%	91.63%		73.38%
	LossDiff *(*${D}_{c}$*)*			**98.81%**	**97.30%**		**89.47%**
Samples discarded by *LossDiff*				6837	9387		10,100

Significant values are in bold.

3 $$Accuracy = \frac{Number\, of\, correctly\, predicted\, labels\, for\, patches}{Total\, number \,of\, patches} \times 100$$

The proposed method achieved state-of-the-art performance for stomach whole-slide images, with patch-based accuracies of 98.81%, 97.30% and 89.47% for the binary, ternary and quaternary classes, respectively (Table 7). These results suggest that the *LossDiff* classification method yields significant improvements in predictive accuracy.

### Confusion matrix analysis

**Figure 7:**
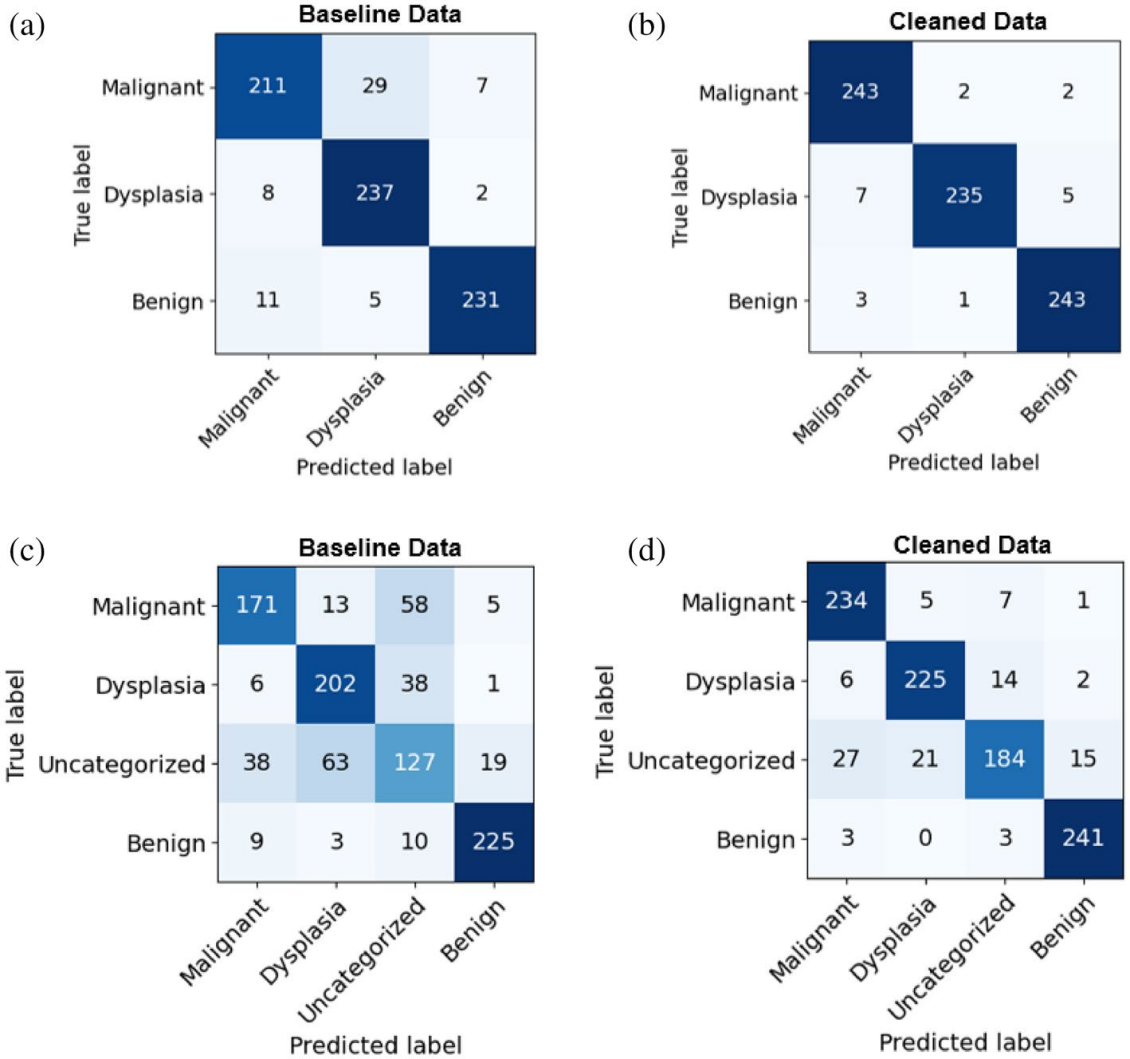
Confusion matrix for ternary classes in the first row (**a,b**) and quaternary classes in the second row (**c,d**).

For medical images, a confusion matrix highlights the key weak points of classification, such as false negatives (Type-II errors). For example, if a patient has a disease and the system generates a false report (i.e., the disease is predicted to be negative for that patient), then the patient may not be diagnosed until the disease reaches an advanced stage, potentially missing the critical window of time for treatment. A confusion matrix enables us to compare the performance of different classes individually. Three positive classes, namely, malignant, dysplasia, and uncategorized, are considered, and they encompass disease diagnoses (positive) that require further assessment; conversely, a benign (negative) diagnosis does not require further evaluation. In the context of this positive vs. negative class distinction, we reduce Type-II errors using the *LossDiff* method. The classification results obtained based on the cleaned data not only exhibit high accuracy but also reduce Type-I and Type-II errors (i.e., $7\to 2$ (see Fig. 7a,b) and $5\to 1$ (see Fig. 7c,d) false negative patches for ternary and quaternary classes, respectively), as shown in Fig. 7. From the confusion matrix analysis, an overall improvement in false positives and false negatives is found, whereby false negatives are of paramount importance because of its direct consequence on medical diagnostic and treatment. As such, they are also discussed in this study.

### Statistical analysis

To further assess and validate our findings, we performed statistical analyses using a McNemar test. The results of the TEST characterized by *p*-values < 0.001 show that the predictions obtained from the LossDiff and baseline methods are highly significantly different.

### Receiver operating characteristic (ROC) curve analysis

**Figure 8:**
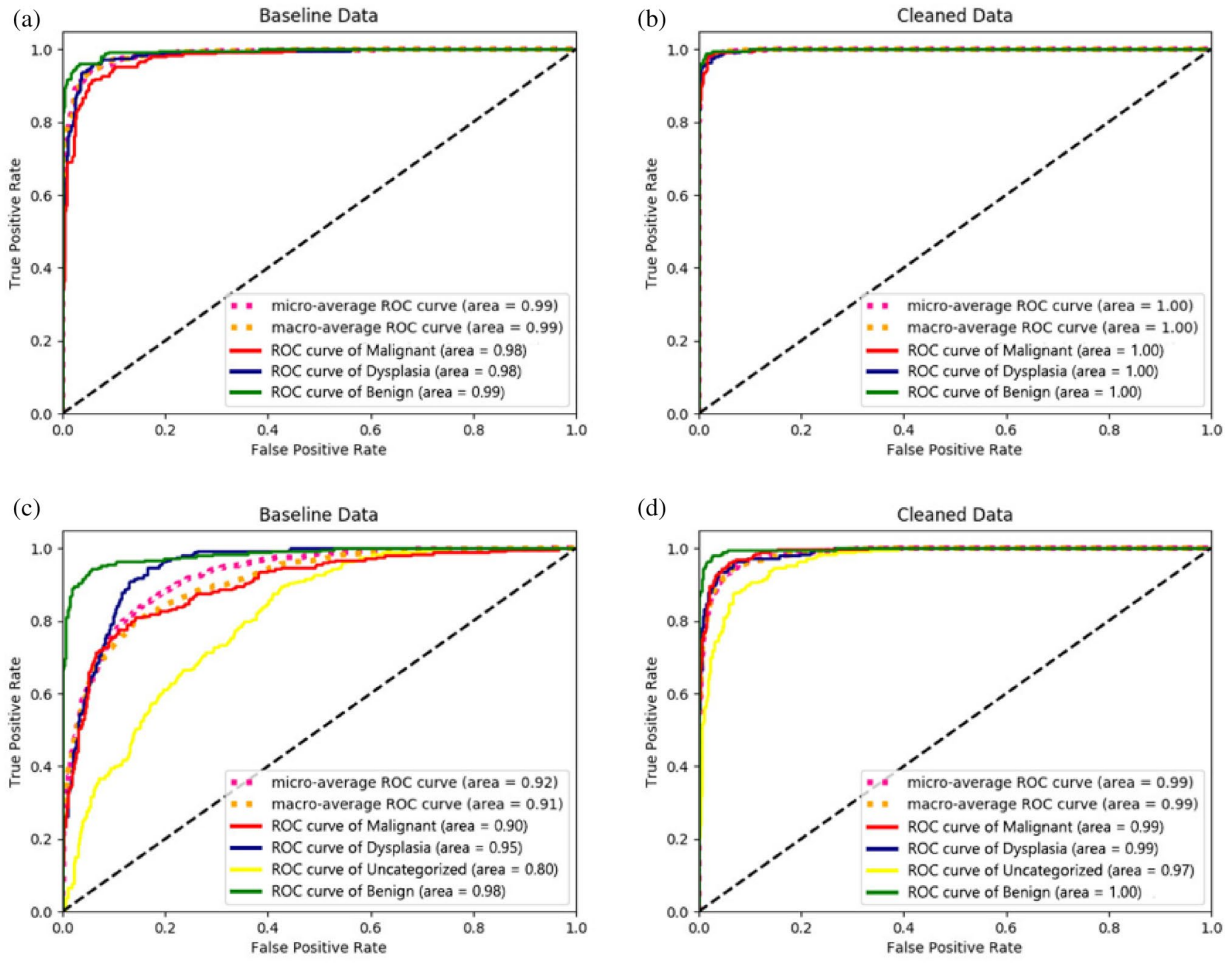
ROC analysis. The first row (**a,b**) shows model performance for ternary classes, and the second row (**c,d**) shows the model performance for quaternary classes.

**Figure 9:**
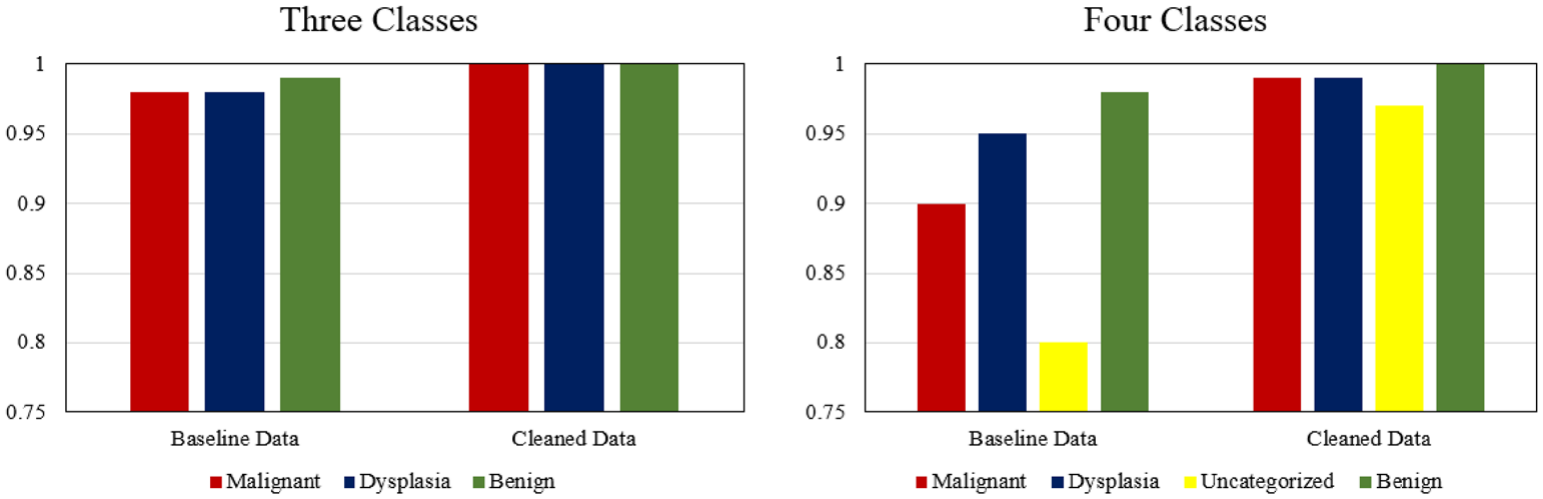
Difference in the area under the ROC curve for the baseline (${D}_{b}$) and cleaned data (${D}_{c}$).

In addition to the confusion matrices used to compare the performance of the methods for different classes, an ROC analysis was performed as a critical evaluation used for medical diagnostic systems^59^. We analyzed the ROC curves to determine the true-positive rate and false-positive rate of patches. Figure 8 shows that the model achieved a significant improvement in ROC when the cleaned data (obtained via *LossDiff*) were used. The micro-average ROC curve, computed from the sum of all true positives and false positives across all classes, shows improvement for the model trained on cleaned data (see Fig. 8b–d). The macro-average ROC curve, computed using an average of curves across all classes, also shows improvement for the model trained on cleaned data (see Fig. 8b–d). Figure 9 further shows the exact difference in the area under the ROC curve between the baseline (see Fig. 8a–c) and cleaned data (see Fig. 8b–d).

### Feature space visualization analysis

**Figure 10:**
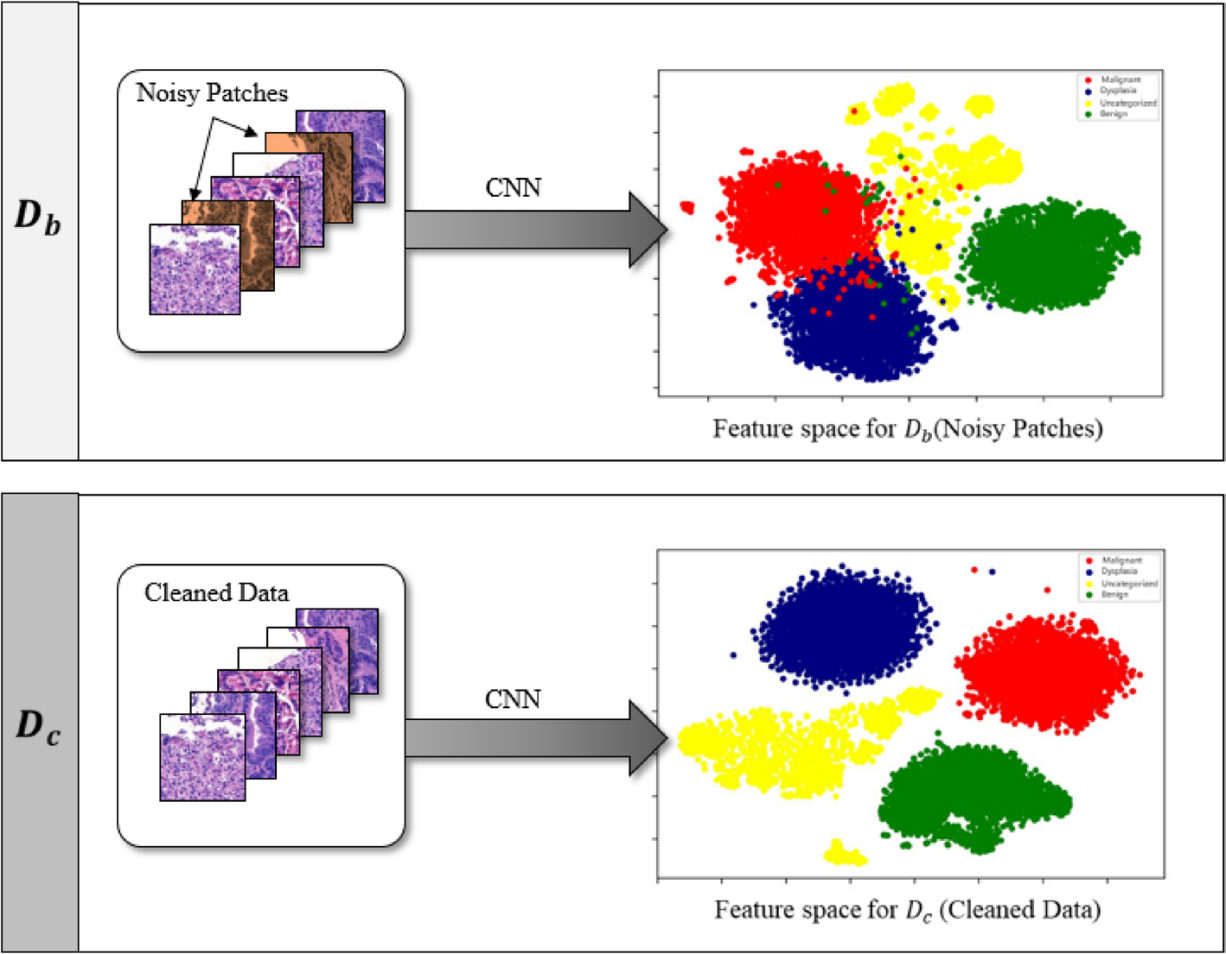
Feature space visualization for DenseNet-201 features using *t*-SNE dimensionality reduction based on baseline and cleaned data. The red, blue, yellow, and green colors denote the, malignant, dysplastic, uncategorized and benign classes, respectively.

It is often challenging to visualize a high-dimensional feature space. Thus, we used the *t*-SNE dimensionality reduction technique to validate model performance by visualizing the feature space. The model features are extracted using a model trained on both baseline and cleaned dataset patches. This analysis aimed to show the difference between the feature spaces of the two models. Hence, we have simply used the default parameters of the scikit-learn *t*-SNE method. Figure 10 shows that the feature space for the baseline is relatively scattered and classes overlap with each other; however, the feature space for the cleaned data is well confined, and classes are clearly separated, implying that the CNN model yields a well-defined feature space for the cleaned data compared to that for the noisy data.

### Noise handling ability analysis

**Figure 11:**
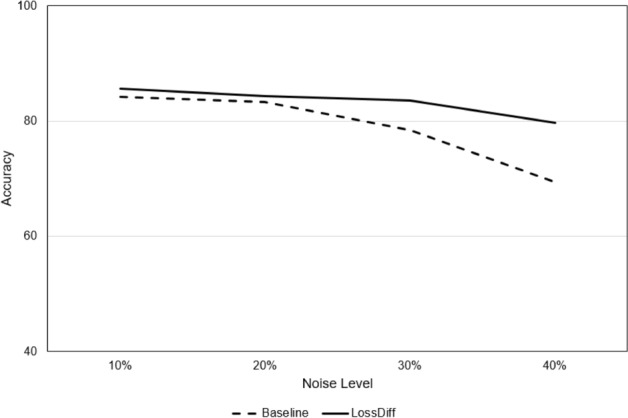
Accuracy comparison between baseline and *LossDiff* at different levels of noise.

We validated the performance of the proposed method by adding synthetic noise to a publicly available dataset. Synthetic noise is applied randomly by changing the labels to the opposite class in each distribution by various percentages (10%, 20%, 30%, and 40%). Our results for varying noise levels further underscore the robustness of the proposed method (*LossDiff*), even with high noise levels; notably, *LossDiff* exhibited 10% better accuracy than the baseline method for 40% synthetic noise, as shown in Table 8. Figure 11 also shows that *LossDiff* is more robust than the baseline model at different noise levels. To mitigate noise, two sets of configurations were adopted: sample discarding and label flipping. Sample discarding yielded better results than label flipping. One of the main causes of the improved performance using sample discarding may be the removal of uncertain labels. If we perform label flipping, many misclassifications increase model complexity and negatively influence convergence. It is also worth noting that for extensive noise levels, label flipping occurs more than sample discarding because the model attempts to converge based on newly flipped data.

**Table 8:** Accuracy comparisons for different noise levels between the baseline method (with label noise) and *LossDiff* (without label noise) for sample discarding and label flipping approaches.

Measure	Configuration	Percentage of noise			
		10	20	30	40
Accuracy	Baseline	84.23	83.31	78.45	69.33
	*LossDiff* (Sample discarding)	85.59	**84.27**	**83.51**	**79.67**
	*LossDiff* (Label flipping)	**85.75**	84.09	81.31	77.13
Number of samples affected	Samples discarded by *LossDiff*	26,178	**52,376**	**78,243**	**104,770**
	Samples flipped by *LossDiff*	**21,271**	53,779	85,253	113,565

Significant values are in bold.

### Comparison with the related work

To demonstrate the superiority of the proposed method, we have compared our method with the competing methods from the literature, which focus on label noise (see Table 9). To the best of our knowledge, this study is among the first to assess and report the results of different label denoising methods for whole-slide images. Note that the details of competing methods can be found in their respective studies^60–64^ and as such, their detailed descriptions are omitted from this study.

**Table 9:** Accuracy comparisons between extant label noising methods and *LossDiff* using the same set of stomach image data.

Classes	Malignant and benign (binary)	Malignant, dysplasia, and benign (multiple ternary)	Malignant, dysplasia, uncategorized, and benign (multiple quaternary)
Mixup^60^	98.61	91.23	76.16
Co-teaching^61^	93.72	88.30	71.25
Deep abstaining classifier^62^	98.59	95.14	77.18
Symmetric cross-entropy loss^63^	95.74	91.90	72.57
Confidence learning^64^	93.51	89.87	70.70
LossDiff	**98.81**	**97.30**	**89.47**

Significant values are in bold.

**Figure 12:**
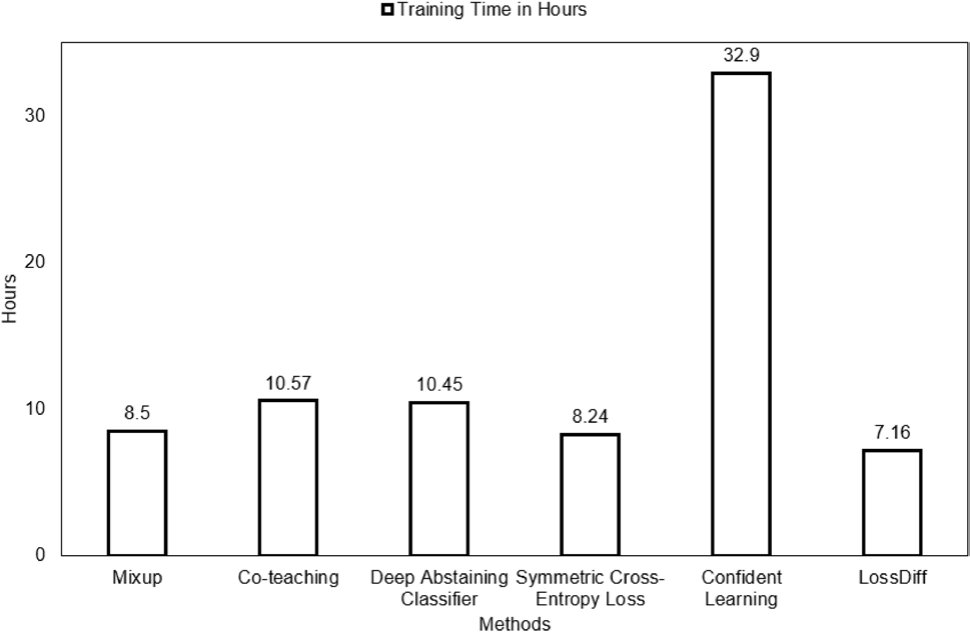
Training time comparison for different noise reduction methods.

We first evaluated these methods using their default hyperparameters and then used settings similar to those in *LossDiff*. Note that all methods were tested on the same balanced data to avoid the bias associated with easy-to-classify patches and certain distributions. Two methods, the deep abstain classifier and confidence learning methods, use a filtering approach; both these methods were tested on the cleaned data generated from these methods and the proposed method. Four methods, i.e., baseline, Mixup, co-teaching, and symmetric cross-entropy loss, were tested based on the baseline test data and cleaned data generated by the proposed method. The training times for different methods are reported in Fig. 12, which shows that *LossDiff* is efficient in terms of time complexity.

As shown in Table 9, *LossDiff* outperforms all other methods, including the deep abstaining classifier, which is the second-best performer. Our proposed *LossDiff* method monitors the loss of correctly classified instances only in batches rather than considering all cases at once. This approach mitigates overfitting by eliminating the samples with loss values higher than the average loss in all iterations, even if they are correctly classified, thereby reducing the likelihood of overfitting.

Note that the each model could be improved by adjusting the values of hyperparameters, but due to space constraints, we report the best results for the two considered configurations. *LossDiff* requires the shortest training time for two reasons. First, decisions regarding noise predictions are simple, as described in the Methods section. Second, *LossDiff* uses a sample discarding approach that eliminates uncertain data, making the training dataset small but adequate.

## Discussion

Whole-slide image analysis is the gold standard for diagnosing different types of cancers. The prevalence of stomach cancer is high among various types of cancers^48^. As such, there is a need for automated diagnostic systems for assessing whole-slide images of stomach cancer. Notably, conventional machine learning algorithms are not suitable for identifying and predicting complicated patterns of digital pathology, which poses several challenges^4^,^37^ for deep learning. Specifically, challenges such as the requirement of a large training dataset, the curse of dimensionality, and labeling a large amount of data hinder the practical applicability of CNNs to whole-slide images of cancer in general and stomach cancer in particular.

Digital pathology aims to eliminate the requirement of large amounts of training data by providing ease of data access for different networks, thus enabling researchers to use data remotely and instantly share information^4^. Whole-slide images contain gigapixels of data, whereas CNNs usually process images of small size because of computational limitations. Most researchers use a patch-based classification for whole slide images^5^ using CNNs. One of the ignored problems with regard to whole-slide image analysis is weakly annotated data, which is practically unavoidable, as it is almost impossible for a human annotator to create a precise pixel-level segmentation result when labeling a problematic area. Most abnormal annotations include small benign regions, thus resulting in label noises (or false positives) in the training data. To resolve label noise issues in the training data, past research has focused on benchmark datasets related to distinguishable objects and medical images, whereas whole-slide images have largely been ignored.

To overcome patch-based label noise problems, this study presents a method called *LossDiff* for filtering and removing patch-based label noise. Initially, we consider the loss of correctly classified labels and compare the corresponding value with the average batch loss. In this way, a CNN can learn the general distribution of loss up to a specific number of epochs. The CNN then starts filtering samples if the minibatch loss surpasses the average batch loss. This method does not require any subset of cleaned samples for training, unlike mentor and co-teaching approaches^10,61^. The proposed method also avoids the need for an extra layer of hidden units, additional classes, and multiple loss functions to learn the noise distribution^39,43,47^. The targeted and straightforward nature of the proposed method enables it to mitigate patch-based label noise by providing an adequate and effective solution for leveraging data, time, and computational resources.

**Figure 13:**
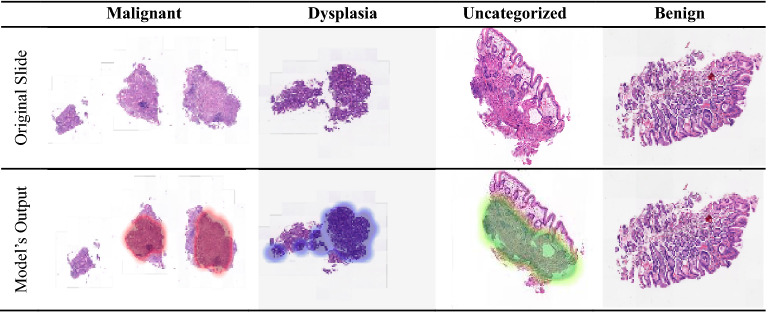
The final output of the CNN trained based on the cleaned malignant, dysplasia, uncategorized, and benign data and the corresponding heatmaps of abnormal regions.

To validate the performance of the proposed approach, several evaluation methods were employed, and notable improvements were achieved with the cleaned data. *LossDiff* yielded an accuracy of 98.8%, with an approximately 4% improvement over the baseline, for the binary classification problem, 97.3% accuracy, with an approximately 6% improvement over the baseline, for the ternary-class problem, and 89.5% accuracy, with an approximately 15% improvement over the baseline, for the quaternary-class problem. Additionally, the confusion matrix shows decreases in false negatives and false positives, which are critical for diagnostic systems; notably, false negative diagnoses can have significant adverse implications for patients’ proper treatment plans and survival chances. The results of the test characterized by *p*-values < 0.001 show that the predictions obtained from the LossDiff and baseline methods are highly significantly different.The area under the ROC curve for the clean data obtained via *LossDiff* also displays a substantial improvement in the true-positive rate versus the false-positive rate compared to that for the original data. Feature space visualization using *t*-SNE further validates the performance of the proposed approach, and the CNN produces a much better confined feature space with the cleaned data than with the baseline data. One important thing to note from the feature space visualization results is the uncategorized class, which consists of abnormalities (specifically, atypical glandular proliferation, neuroendocrine tumors, submucosal tumors, low-grade lymphoma, and stromal tumors). These subgroups not only add intraclass complexity but also affect the model’s performance (see Fig. 13). Thus, we evaluated ternary and quaternary classes separately. We also checked the model robustness using several noise levels, and the results show that the model is robust, even at high noise levels, as reported in Table 8. To demonstrate the final model output, we present the CNN results in Fig. 13, which shows the heatmaps of abnormal regions next to the input slides.

In the past, several studies employed different techniques to improve the classification of whole-slide images (see Table 10). Until 2015, researchers focused on handcrafted feature extraction techniques, which required additional human effort and were unreliable given varying environmental factors such as lighting conditions, different microscopes, and staining methods. CNNs, however, can automatically extract useful latent features and provide better generalization results for unseen data^65^. Many of the studies of whole-slide images have considered different machine learning classification models and ignored the label noise problem. In this regard, the proposed method can improve the applicability of CNNs in whole-slide image analysis by systematically mitigating the label noise issue. In terms of performance improvement, the proposed method yields notable outcomes by explicitly considering the label noise issue (see Table 7).

**Table 10:** Methods and results for computer-aided analyses of whole-slide stomach images.

Study	Objective	Feature selection	Technique
Sharma et al.^66^	Leukocytes, epithelial nuclei, fibrocytes/border cells, other nuclei classification	Handcrafted	AdaBoost classification
Sharma et al.^67^	Feature extraction and Nontumor, Her2/neu + tumor, Her2/neu-tumor classification	Handcrafted	Relational graphs
Sharma et al.^68^	HER2 + tumor, HER2 − tumor, and Nontumor classification	Automated	CNN
Qu et al.^69^	Epithelium, stroma, and tissue background classification	Automated	CNN fine tuning
Li et al.^70^	Malignant and benign classification	Automated	CNN
Kim et al.^71^	Malignant region, tubular adenoma (TA), and benign classification	Automated	CNN and random forest classifier
Wang et al.^72^	Malignant, dysplasia, and benign classification	Automated	Multi-instance learning using a CNN
Song et al.^73^	Malignant and benign classification	Automated	DeepLab v3 segmentation for slide-level classification

We evaluated the performance of recently published methods of label noise removal based on whole-slide image data and found that *LossDiff* provides the best results (see Table 9).

One of the possible reasons for the higher accuracy of the proposed method compared to previous methods can be attributed to the focus on individual classes and the comparison of the overall loss distribution for correct predictions versus the loss distribution of correctly classified instances within a batch. Correctly classified instances with high loss can result in overfitting, as shown in Fig. 5, but *LossDiff* systematically eliminates such samples. Moreover, *LossDiff* continuously filters and removes noisy patches during the training phase, allowing the CNN to be retrained on a new version of data every epoch. Rather than inputting the corrupted labels into the CNN again, the network uses the data that have been filtered. Another advantage of this approach is that it does not rely on verified data^46^ or co-teaching approaches^61^. Our results indicate that reducing patch-based label noise before performing cancer classification based on whole-slide images can significantly enhance model performance. Enabling the model to learn the cell morphology instead of relying on the forced memorization of patches yields improved classification performance. Training based on cleaned data over time aids in model calibration compared to using data with noisy labels, as shown in Fig. 10.

In a future study, the threshold $\alpha$, which was set empirically in this study to avoid the elimination of difficult cases (with true positives), can be learned by adding a layer of learnable parameters in parallel to the existing architecture. Another future research direction is to analyze filtered patches in detail, which can help avoid the possibility of filtering true positive patches and aid the system in saving training data by not filtering patches with correct labels and improve model performance by leveraging the most-useful training data.

In conclusion, the morphology of whole-slide images makes the labeling process vulnerable to human error, resulting in false-positive regions, which exacerbate the automated detection of cancer at the patch level. Noisy patches in whole-slide images can affect CNN performance, as the model may struggle to converge in the presence of label noise. In this study, we proposed a deep learning patch label denoising method (*LossDiff*) to eliminate noisy patches from whole-slide images. *LossDiff* eliminated the need for extra layers in capturing the noise distribution and reduced the reliance on predefined verified labels and curriculum-like approaches. The performance comparisons of the proposed method with competing methods using the same dataset of whole-slide images showed that *LossDiff* yielded the best patch-level accuracy. A McNemar test further statistically validated and confirmed the difference between *LossDiff* and the baseline methods. With a publicly available dataset and various levels of induced synthetic noise, *LossDiff* also showed superior performance. Given the high cost of producing explicit annotations for whole-slide images and the unavoidable error-prone nature of human annotations of medical images, the proposed method has practical implications for whole-slide image annotations and automated cancer diagnosis. This approach can save time and money in generating clean sets of training data and provide improved classification results, ultimately enhancing patient treatment plans and survival chances.

## Data Availability

The stomach whole-slide images used in this study were collected by Seegene Medical Foundation, South Korea. Data are not available for public use, and restrictions apply. Detailed information about data collection and processing is provided in the Dataset subsection. The public dataset used in this study is available^51^.
